# Parenteral nutrition associated cholestasis

**DOI:** 10.1186/1824-7288-41-S1-A4

**Published:** 2015-09-24

**Authors:** Simonetta Costa, Giovanni Barone, Piero Catenazzi, Costantino Romagnoli

**Affiliations:** 1 Department of Paediatrics, Division of Neonatology, Catholic University of Sacred Heart, Rome 00168, Italy

## 

Parenteral nutrition (PN) is life saving for many preterm infants and other neonates with severe illness, but prolonged use of PN can lead to intrahepatic cholestasis, referred to as parenteral nutrition–associated cholestasis (PNAC). It is defined as direct bilirubin greater than 2.0 mg/dL persistent for at least 2 consecutive tests duringthe administration of PN, not associated with other known causes of cholestasis [1-3].

With the increasing survival of preterm infants and neonates requiring intensive care, PNAC has become a more common clinical challenge. The incidence of PNAC varywidely depending on the population studied, with high incidencein populations carrying several risk factors for PNAC. It increases with duration of PN and ranges from 10% to 85% in infants [4-8].

A multifactorial aetiology has been proposed for the development of PNAC. Recognized risk factors for PNAC include low birth weight, low gestational age, necrotizing enterocolitis, intestinal malformations, and intestinal surgery. A further risk factor is the occurrence ofsevere infections, due to the requirement for central line for infusion of PN, and bacterial overgrowth caused by enteralstarvation and immature immune function[9-13].

However, exposure to PN is demonstratedas the main factor in the development of PNAC. Intravenous hyper-alimentationhas been implicated, such as thetotal caloric overload, the quality of aminoacid solutions, the cumulative amount and the quality of lipid infusion, the presence of excessivealuminium in the PN solution, and the high manganese intake with PN [1,14-17].

Ursodeoxycholic acid, cyclic PN, light protection for PN, tapering the soybean-based lipid emulsion, and antibiotics to decontaminate bacterial overgrowth are used to treat PNAC [18-21].

In recent years, increasing attention has been paid to the lipid content in PN. It has been found that fish oil–containing lipid emulsions could be useful in infants to reverse PNAC for whom enteral feeding is intolerable. However, no evidence supports the use of fish oil–containing lipid emulsions to prevent PNAC in neonates, including preterm infants [22].

Enteral feeding remains the best strategy to reverse and prevent PNAC, with as little as 10% of caloric intake showing beneficial effects [5,22].
